# Fermented *Astragalus* Powder, a New Potential Feed Additive for Broilers to Improve the Growth Performance and Health

**DOI:** 10.3390/ani14111628

**Published:** 2024-05-30

**Authors:** Songwei Han, Guowei Xu, Kang Zhang, Saad Ahmad, Lei Wang, Fubin Chen, Jiahui Liu, Xueyan Gu, Jianxi Li, Jingyan Zhang

**Affiliations:** 1 Lanzhou Institute of Husbandry and Pharmaceutical Sciences, Chinese Academy of Agricultural Sciences, Lanzhou 730050, China; hansongwei0@163.com (S.H.);; 2 Cell Biology and Immunology Group, Wageningen University & Research, 6708 WD Wageningen, The Netherlands

**Keywords:** fermentation, traditional Chinese medicine, immune function, antioxidant capacity, intestinal microbiota

## Abstract

**Simple Summary:**

*Astragali* Radix cell wall is composed of dense components, which greatly reduces the utilization rate of its active constituents and limits its application and promotion in animals. The application of microbial fermentation technology can effectively address this issue. We produced a mixed-bacterial-fermented *Astragalus* powder. In this study, we found that fermented *Astragalus* powder has the potential to enhance the growth performance, immune function, and antioxidant capacity of broilers to a certain degree. Additionally, it has shown promising effects in regulating the intestinal flora. Fermented *Astragalus* powder is expected to be a novel kind of feed additive.

**Abstract:**

A total of 320 1-day-old broilers were randomly divided into five groups. The control group (CON) received a basal diet, while the FAP4, FAP2, and FAP1 groups were provided with the basal diet supplemented with 4%, 2%, and 1% fermented *Astragalus* powder, respectively. The unfermented *Astragalus* powder (UAP2) group was fed the basal diet supplemented with 2% UAP. Each group contained eight replicates of eight chicks each. The results revealed that the final BW and ADG in the FAP 1 and FAP2 were higher than those in the UAP2 and CON groups, while reducing F/G from day 14 to day 42. On day 42, the thymus index in the UAP and FAP groups as well as the bursa index in the FAP4 group showed significant increases compared to those in the CON group. Supplementation with 2% FAP elevated serum IgA levels in broilers on day 28 and day 42, and it also increased serum IgG levels on day 42. Furthermore, supplementation with 2% FAP elevated serum albumin (ALB) levels in broilers, while supplementation with 4% FAP increased serum (glucose) GLU levels in broilers on day 28. The serum biochemical parameters and pathological observation of the liver and kidney in the groups did not show any adverse effects on broilers’ health. In addition, the serum total antioxidant capacity (T-AOC) level significantly increased in the FAP4 and FAP2 groups on day 28, and the malondialdehyde (MDA) level in both serum and liver tissue decreased in the FAP2 group on day 28 and day 42. Compared to the CON group, 2% FAP and 2% UAP supplementation reduced the relative abundance of *Bacteroides* and supplementation with 2% FAP increased the relative abundance of *Alistipes* on day 42. In conclusion, the dietary supplementation of FAP can enhance the growth performance, immune function, and antioxidant capacity and regulate microflora in broilers, of which 2% FAP is more effective. It indicates FAP exhibits significant application potential as a promising feed additive for broilers.

## 1. Introduction

The projected global population is anticipated to reach 9.6 billion by the year 2050 [1]. To meet the increasing demand for chicken meat, many intensive broiler farms have been established. However, this farming method contradicts the natural growth pattern of broilers, leading to slower growth rates [2]. Simultaneously, high-density breeding environments can cause immunity decline and oxidative stress of broilers [3]. As a result, antibiotic growth promoters are widely used, resulting in problems like intestinal flora imbalance [4], bacterial resistance [5], and antibiotic residues in chicken meat [6]. These issues have significant adverse effects on both poultry and human health. Consequently, the development of safe and effective “natural growth promoters” has become a focal point of current research.

Traditional Chinese medicine (TCM) is expected to become a popular choice as “natural growth promoters“ due to its non-drug resistance and diverse prebiotic functions [7]. *Astragalus* is the dried root of *Astragalus membranaceus* (Fisch.) Bge. var. *mongholicus* (Bge.) Hsiao or *A. membranaceus* (Fisch.) Bge. and is used as a traditional herb in China, USA, Japan, Korea, Iran, Russia, and some other European countries [8]. It contains polysaccharides [9], saponins [10], flavonoids [11], and other active ingredients. *Astragalus* has been widely used in poultry, with functions such as elevating immunity and antioxidation and regulating intestinal microflora [12]. However, there are a lot of lignin, cellulose, and pectin in Astragalus, which are closely bound with bioactive substances, and these substances inhibit the release of bioactive substances in Astragalus to the external environment [13]. Probiotic fermentation presents a potential solution to this issue. The process of microbial fermentation generates a complex enzymatic system that not only facilitates the release of active ingredients from *Astragalus* [14], but also catalyzes the degradation, modification, or transformation of these active ingredients into more potent bioactive compounds [15].

In our previous study, a fermentable strain of *Streptococcus alactolyticus* was isolated [16] from chicken cecum and evaluated for safety [17]. Further investigation showed that liquid fermentation of *Astragalus* using *S. alactolyticus* significantly increased the levels of polysaccharides and flavonoids [18,19]. Considering that the product of multi-strain mixed fermentation is richer in nutrients and active ingredients than that of single-strain fermentation and also being more conducive to animal growth and feed utilization [20,21,22], we established and optimized the liquid fermentation process of *Astragalus* fermentation with mixed bacteria (including *Lactobacillus acidophilus*, *Lactobacillus reuteri*, and *S. alactolyticus*). Currently, there has been no systematic appraisal of the application of *Astragalus* fermented by mixed bacteria as a feed additive for broilers. Therefore, this study focused on the effects of FAP on the growth performance, immune function, antioxidant capacity, and intestinal microbiota in broilers, providing evidence for the application of mixed-bacteria fermentation technology in *Astragalus* and promoting the use of FAP as new feed additives in broilers.

## 2. Materials and Methods

### 2.1. Fermentation Strains and Herbs

The fermentation strains were the mixture of *L. acidophilus*, *L. reuteri*, and *S. alactolyticus* with a ratio of 1:1:1, with a cell density of 1 × 10^8^ CFU/mL. *L. acidophilus* and *L. reuteri* were provided by North Na Biotechnology Co., Ltd. (Langfang City, Hebei Province, China). *S. alactolyticus* (GenBank accession No. JX435470; China Patent No. 20120141827.5) was derived from the chicken cecum, isolated, and preserved in the Veterinary Laboratory of Lanzhou Institute of Husbandry and Pharmaceutical Sciences of CAAS. *Astragalus* Radix was purchased from Gansu Huanghe Pharmacy Market (Lanzhou, China) and identified by Agricultural Product Quality Inspection & Safety Test Center of Gansu Province (Lanzhou, China).

### 2.2. Preparation of Fermented Astragalus Powder

Fermented *Astragalus* powder (FAP) and unfermented *Astragalus* powder (UAP) were prepared by Lanzhou Institute of Husbandry and Pharmaceutical Sciences of CAAS. The preparation of FAP involved adding 6% of the mixture of bacterial suspensions into an aseptic liquid fermentation medium containing *Astragalus*, glucose, and other materials. The mixture was evenly mixed and subjected to anaerobic fermentation at 37 °C for 42 h. Afterwards, corncob powder was added to the fermented *Astragalus* and mixed thoroughly. Subsequently, the mixture was dried at 70 °C. The dried fermented *Astragalus* was ground into <0.30 mm powder, to obtain FAP. The preparation of UAP followed a similar process to that of FAP, except that the mixture of bacterial suspensions was replaced with sterile MRS broth (bacterial culture medium). The main nutritional and active components of UAP and FAP were determined through laboratory testing (Table 1).

### 2.3. Animal Treatment and Experimental Design

Based on a single-factor completely randomized design, a total of 320 one-day-old male Sanhuang broilers from a local commercial farm (Lanzhou, Gansu, China) and broiler breeding trials were conducted at this commercial farm (Xigu Family Farm). After a 14-day adaptation period, all birds were randomly assigned into five groups, consisting of 8 replicates with 8 chickens per replicate, which were then denoted as CON group (basal diet), UAP2 group (basal diet supplemented with 2% unfermented *Astragalus* powder), FAP4, FAP2, and FAP1 groups (basal diet supplemented with 4%, 2%, and 1% fermented *Astragalus* powder). The additive and the base diet granules were blended together using a mixer before being fed. The basal diet used in this study was formulated in accordance with the National Research Council (NRC) guidelines to meet the recommended nutritional requirements of broilers (Table 2) and provided to broilers without any anticoccidial or antibacterial supplements. All birds are raised indoors in off-ground sheds, with free feeding and watering. The coop temperature was maintained at 33 °C from 1 to 4 days of age, followed by a weekly decrease of 3 °C. The temperature was maintained at 24 °C until the end of the experiment. Vaccination procedures were carried out as per standard protocols throughout the trial.

### 2.4. Growth Performance

The weekly feed consumption of broilers was recorded for each replicate. Weighing occurred 12 h after feeding cessation at 28 and 42 days of age. Subsequently, the average daily feed intake (ADFI), average daily gain (ADG), and feed-to-gain ratio (F/G) were calculated respectively.

### 2.5. Sample Collection

**Figure 1:**
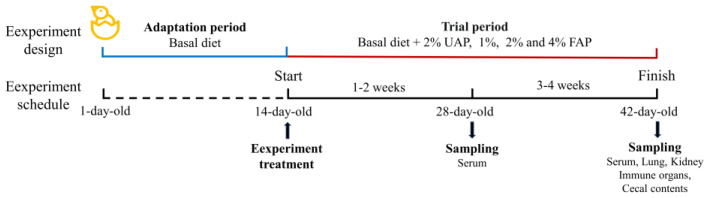
The experimental design and sampling schedule. UAP: unfermented Astragalus powder; FAP: fermented Astragalus powder.

The treatment schedule and sampling protocol is shown in Figure 1. The methods for the sample collection are described below.

At 28 and 42 days of age, broilers were fasted for 12 h prior to individual weighing. Broilers with body weights close to the average of their respective replicates were selected and blood was collected from the wing veins. The serum was then separated and stored at −20 °C.

At 42 days of age, the blood collected broilers were euthanized by bleeding from the jugular vein. Afterwards, the liver, kidney, spleen, thymus, and bursa were removed, and the fat was stripped and weighed. Liver and kidney tissues from the same part of each broiler were fixed with 4% paraformaldehyde for 24 h.

The end of the cecum of collected broilers was cut with sterilized scissors, and the cecum content was collected in a sterile centrifuge tube, temporarily stored in liquid nitrogen, and then transferred to the refrigerator at −80 °C for storage.

### 2.6. Main Organ Indexes

The main organ indexes of the broilers in the groups were calculated according to the formula as follows:

Organ index = immune organ weight (g)/live body weight (kg).

### 2.7. Serum Biochemical Parameters and Immunoglobulin Levels

The levels of serum total protein (TP), albumin (ALB), aspartate aminotransferase (AST), alanine aminotransferase (ALT), creatinine (CREA), urea (UREA), and glucose (GLU) were analyzed by an automatic biochemistry analyzer (Erba XL-640, Mannheim, Germany). The concentrations of Immunoglobulin A (IgA) and Immunoglobulin G (IgG) in serum were determined by ELISA kit (Beijing Solarbio Technology Co., Ltd., Beijing, China).

### 2.8. Histological Analysis

The fixed liver and kidney tissues were dehydrated with varying concentrations of alcohol, paraffin-embedded, and sliced. Finally, the liver and kidney sections were stained with hematoxylin and eosin solution (H&E). The pathological changes in the liver and kidney were observed under the optical microscope (BX43 + DP26, Olympus, Tokyo, Japan).

### 2.9. Antioxidant Capacity

The levels of total antioxidant capacity (T-AOC), glutathione (GSH), and malondialdehyde (MDA) in both serum and liver samples were determined using colorimetric kits (Nanjing Jiancheng Bioengineering Institute, Nanjing, China).

### 2.10. Microflora of Cecum Analysis

Total genomic bacterial DNA was extracted from the cecal contents using the Fecal Genome DNA Extraction Kit (Tiagen Biochemical Technology Co., Ltd., Shanghai, China). Subsequently, the full-length 16S rRNA gene was amplified using the primers (27F: 5′-AGRGTTTGATYNTGGCTCAG 3; 1492R: 5′-TASGGHTACCTTGTTASGACTT-3). PCR products were sequenced on Sequel II after being quantified, repaired, and purified. The sequencing data were processed using SMRT Link (v 8.0) to obtain Circular Consensus Sequencing (CCS). Lima (v 1.7.0) was employed for CCS filtering, and the Effective-CCS was derived by eliminating the Mosaic. Sequences with more than 97% similarity were considered the same operational taxonomic unit (OTU). The OTU sequences were subsequently annotated with reference to the SILVA database using classify-sklearn module in QIIME2 (v 2020.6). Based on the annotation results, differences in species composition were analyzed at the phylum and genus levels by relative abundance histogram. Next, QIIME2 (v 2020.6) was used to evaluate community diversity through α diversity analysis (including ACE, Chao1, Simpson, and Shannon indexes). β diversity was performed based on the weighted unifrac algorithm to compare the overall dissimilarity of cecal bacteria between three group samples and was visualized using principal coordinate analysis (PCoA) and non-metric multi-dimensional scaling (NMDS). The above analyses were performed using BMKCloud (www.biocloud.net).

### 2.11. Statistical Analysis

The data were analyzed by IBM SPSS statistical software (v 26.0). Multiple comparisons were performed using one-way analysis of variance (ANOVA) followed by Tukey’s multiple comparisons test. Statistical plots were generated by GraphPad Prism software (v 8.06). A significance level of *p* < 0.05 was considered statistically significant.

## 3. Results

### 3.1. Growth Performance

The effect of FAP supplementation on the growth performance of broilers is shown in Table 3. From 14 to 42 days, the final BW and ADG were significantly higher in the groups FAP1, FAP2, and FAP4 compared to that in the CON group, with a notable reduction in F/G across all FAP (*p* < 0.05). However, there were no significant differences in final BW, ADG, and F/G between the UAP2 group and the CON group during this period. Between days 14 and 28, as well as days 29 and 42, the ADG in the FAP1 and FAP2 groups was significantly higher than that in the CON group (*p* < 0.05). Additionally, the F/G in FAP1 and FAP2 groups was significantly lower than that in the CON group (*p* < 0.05). However, there were no significant differences observed in both ADG and F/G between the UAP2 group and the CON group (*p* > 0.05). Moreover, the addition of both FAP and UAP had no significant effect on feed intake (*p* > 0.05). The survival rate increased in UAP2, FAP4, FAP2, and FAP1 groups compared to that in the CON group; no significant difference was observed during the whole period (*p* > 0.05).

### 3.2. Main Organ Indexes

The results regarding the effect of FAP on main organ indexes of broilers are presented in Table 4. The thymus index of three FAP groups and UAP2 group was significantly higher than that of the CON group (*p* < 0.05). The thymus index of the FAP2 group was the highest, with a significant difference observed compared to that of the UAP2 group (*p* < 0.05). No significant difference in the spleen index was observed among all groups (*p* > 0.05). However, the bursa index of FAP4 group exhibited a significant increase compared to that of the CON group (*p* < 0.05). Furthermore, there were no significant differences in liver index and kidney index among the groups (*p* > 0.05).

### 3.3. Serum Biochemical Parameters and Immunoglobulin Levels

As shown in Table 5, supplementation with 2% FAP led to elevated serum ALB and IgA levels in broilers at 28 days (*p* < 0.05) compared to the CON group. Additionally, supplementation with 4% FAP increased serum GLU levels in broilers at 28 days (*p* < 0.05). Furthermore, supplementation with 2% FAP significantly increased serum IgA levels in broilers at 28 days (*p* < 0.05) and both IgA and IgG levels at 42 days (*p* < 0.05) compared to the CON group. However, supplementation with FAP and UAP did not have significant effects on other serum biochemical parameters of broilers.

### 3.4. Histological Observation and Organ Index of Liver and Kidney

**Figure 2:**
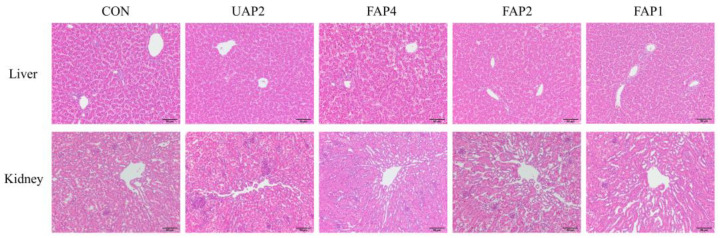
Histopathological changes in liver and kidney in broilers (H&E staining, 200×). CON: control. UAP2, FAP4, FAP2, FAP1 represent that dietary supplementation of 2% unfermented Astragalus powder and 4%, 2%, 1% fermented Astragalus powder, respectively.

The liver lobules of broilers in each group exhibited clear and intact structures, with liver cells arranged radially around the central vein, displaying normal morphology and structural integrity. Similarly, the renal structure of broilers in each group exhibited intact morphology, with clearly visible internal veins of lobules and normal surrounding cortical thin tissue (Figure 2).

### 3.5. Antioxidant Capacity

Compared to the CON group, both FAP2 and FAP4 groups significantly augmented serum T-AOC levels at 28 days (*p* < 0.05). However, there was no significant differences in serum GSH levels among all groups (*p* > 0.05). Additionally, the serum MDA levels in FAP2 group were significantly lower than those in the CON group, UAP2 group, and FAP4 group (*p* < 0.05). Furthermore, at 42 days, the serum MDA levels in FAP2 group were significantly lower than those in the CON group (*p* < 0.05). Conversely, UAP supplementation had no effect on the antioxidant capacity of broilers at 28 and 42 days (*p* > 0.05) (Table 6).

### 3.6. Microflora in Cecum

**Figure 3:**
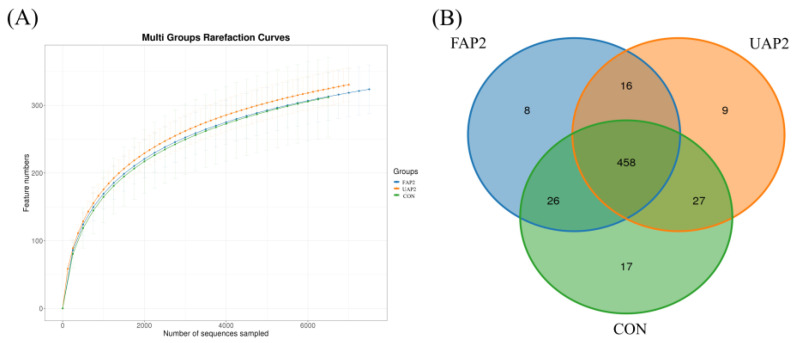
Dilutive curve (**A**) and Venn diagram (**B**). CON: control. UAP2, FAP4, FAP2, FAP1 represent that dietary supplementation of 2% unfermented Astragalus powder and 4%, 2%, 1% fermented Astragalus powder, respectively.

Based on the dilution curve (Figure 3A), it is evident that the curve gradually plateaued, suggesting that the sample sequence was adequate and that the species diversity would not undergo significant increase with further sequencing depth. A total of 214,059 effective CCSs were obtained from the 18 samples, resulting in the identification of 561 operational taxonomic units (OTUs) at a similarity threshold of 97%. The Venn diagram (Figure 3B) illustrated 458 OTUs shared among all the three groups, with the CON group exhibiting a higher count of 528 OTUs compared to the other groups. Moreover, the CON group exhibited 17 unique OTUs, while the UAP2 and FAP2 groups had 9 and 8 unique OTUs, respectively.

**Figure 4:**
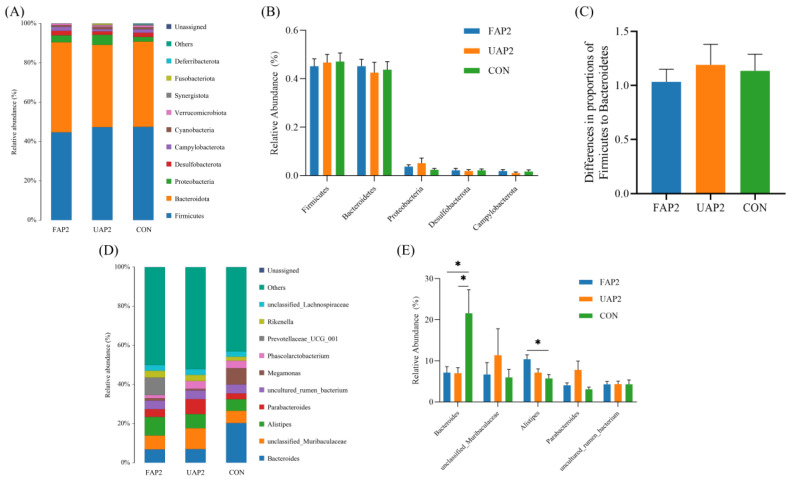
Effects of fermented Astragalus powder on cecal microflora composition in broilers. (**A**) The cecal microflora composition and abundance of each group at phylum level. (**B**) Relative abundance of the five most predominant cecal microflora at phylum level. (**C**) The ratio of Firmicutes to Bacteroidetes. (**D**) The cecal microflora composition and abundance of each group at genus level. (**E**) Relative abundance of the five most predominant cecal microflora at the genus level. All data were evaluated as mean ± SEM (*n* = 6). * *p* < 0.05. CON: control. UAP2, FAP4, FAP2, FAP1 represent that dietary supplementation of 2% unfermented Astragalus powder and 4%, 2%, 1% fermented Astragalus powder, respectively.

The relative abundance of three distinct microflora groups was analyzed at both the phylum and genus levels. At the phylum level (Figure 4A), *Firmicutes* and *Bacteroidetes* were the dominant phyla of all groups, collectively accounting for 90.10% of the total abundance. Additionally, *Proteobacteria*, *Desulfobacterota*, *Campylobacterota*, and *Cyanobacteria* were present in proportions exceeding 1%. Notably, no significant variation in bacterial abundance was observed among all groups (*p* > 0.05). The ratio of *Firmicutes* to *Bacteroidetes* (F/B), being an indicator of microbial imbalance [23], remained unaffected by treatments (Figure 4C). At the genus level (Figure 4D), three types of microflora exhibited relative abundances exceeding 5%, namely *Bacteroides* (11.36%), *unclassified muribactaceae* (8.99%), and *Alistipes* (7.53%). Compared with the CON group, supplementation with 2% FAP and 2% UAP resulted in a significant reduction in the relative abundance of *Bacteroides* (*p* < 0.05) (Figure 4E), while addition of 2% FAP led to an increased relative abundance of *Alistipes* (*p* < 0.05) (Figure 4E).

**Figure 5:**
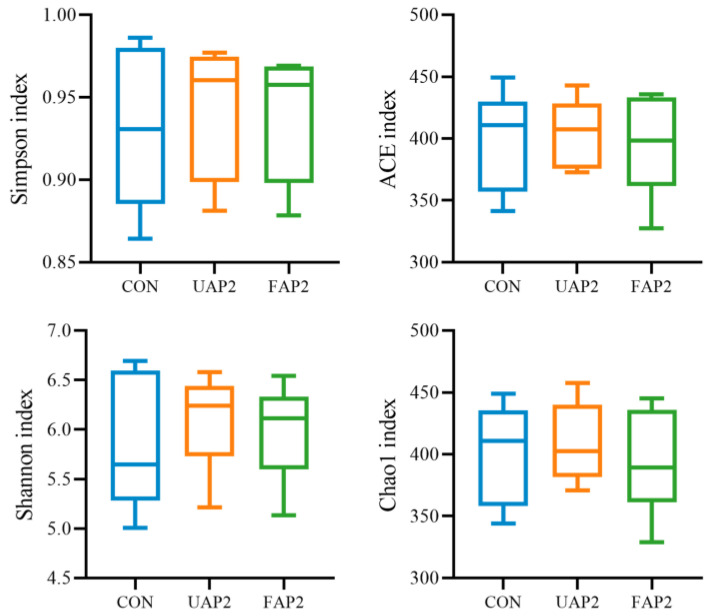
Effect of fermented Astragalus powder on α diversity of cecal microflora in broilers. CON: control. UAP2, FAP4, FAP2, FAP1 represent that dietary supplementation of 2% unfermented Astragalus powder and 4%, 2%, 1% fermented Astragalus powder, respectively.

**Figure 6:**
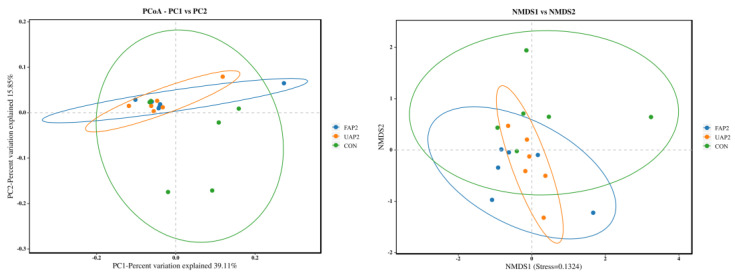
Effect of fermented Astragalus powder on β diversity of cecal microflora in broilers. CON: control. UAP2, FAP4, FAP2, FAP1 represent that dietary supplementation of 2% unfermented Astragalus powder and 4%, 2%, 1% fermented Astragalus powder, respectively.

The α diversity (including ACE, Chao1, Simpson, and Shannon indexes) serves as a measure of the species richness and evenness within intestinal microflora. As illustrated in Figure 5, there is no significant difference in the impact of treatments on the Chao1, Ace, Shannon, and Simpson indexes. However, PCoA and NMDS revealed that the FAP2 and UAP2 groups had a different microbiota structure compared to the CON group (Figure 6).

## 4. Discussion

The fermentation of TCM involves a biological transformation process. Under the action of microorganisms, the original properties of the medicine are enhanced or new therapeutic effects are produced, which may be attributed to extracellular enzymes such as cellulase and pectinase produced by microorganisms breaking the cell wall of TCM and exposing the active ingredients [24]. Therefore, fermentation is one of the effective means to improve the utilization rate of TCM. Tang et al. fermented Lychee pulp with a combination of bacteria, significantly increasing the release of catechins and quercetin. Moreover, the fermented lychee phenolics were found to be more beneficial for the growth of gut microbiota [25]. Similarly, Wang et al. indicated that dietary supplementation with 3% fermented *Artemisia argyi* significantly enhanced the growth performance and meat quality of broilers [26]. Further, Liu et al. also showed that both fermented and unfermented *Andrographis paniculata* exerted positive impact on the growth performance, immune status, and intestinal morphology of ducks, with a more pronounced effect using fermented *Andrographis paniculata* [27].

In poultry farming, growth performance serves as a direct indicator for assessing animal health and the effectiveness of additives. There have been many reports on the study of TCM as growth promoters in broilers. In a study by Liu et al., the inclusion of 1000 mg/kg polyherbal mixtures in the diet of yellow-feathered broilers exhibited a significant enhancement in growth performance and immune status, improvised by improved antioxidant capacity and intestinal function [28]. The results from the study of Zhang et al. showed that *Lasia spinosa* Thw. exerts a discernible growth-promoting effect on broilers [29]. TCM is often characterized by poor taste, which can be improved through fermentation. This enhances its palatability and facilitates its utilization as a feed additive, thereby promoting the incorporation of TCM in animal production practices. Ding et al. discovered that fermented mulberry leaf powder supplementation enhances nutrient digestion and absorption of broilers, thereby promoting growth performance [30]. Wu et al. showed that fermentation had a beneficial effect on the chemical composition of rapeseed meal, leading to increased ADG and ADFI in broilers when fed with fermented rapeseed meal [31]. Building upon this, our study investigated the impact of dietary supplementation of FAP on the growth performance of broilers. From 14 to 28 days, supplementation with 1% FAP exhibited the most significant growth-promoting impact with the highest feed conversion rate. From 29 to 42 days, 2% FAP supplementation showed the best effect on broiler growth. These findings suggest that as broilers gets older, higher FAP dosages are needed to enhance growth. Additionally, the supplementation of UAP can moderately enhance the growth performance of broilers, though without statistical significance. The growth-promoting effects of FAP are likely attributed to increased levels of its active ingredients (Table 1). Young broilers fed diets containing *Astragalus* polysaccharides exhibited a significantly higher weight gain [32]. Flavonoids also can positively regulate the hypothalamic–pituitary–adrenal axis, thereby promoting growth hormone (GH) and insulin-like growth factor (IGF-1) [33]. From the perspective of economic benefits, although the addition of FAP will lead to an increase in feed costs, FAP promotes the growth of broilers, which makes up for this loss, and the use of FAP also improves the survival rate of broilers. Therefore, the supplementation of FAP in broiler diets can improve the economic efficiency of chicken farms. Therefore, *Astragalus*, particularly after probiotic fermentation, demonstrates significant potential as a growth promoter for broilers.

The immune organ index is crucial for assessing the development of animal immune organs, usually used to reflect the immune function and health status of animals [34]. In chickens, the primary immune organs include the thymus, spleen, and bursa, the latter being unique to avian species. These organs serve as crucial sites for the differentiation and maturation of T and B lymphocytes, playing a pivotal role in mounting an effective immune response within the organism [35]. This study unveiled that supplementation with 1% FAP, 2% FAP, 4% FAP, and 2% UAP led to significant increases in the thymus index of broilers compared to the CON group. Notably, the highest thymus index was observed in the FAP2 group. Dietary inclusion of 2% UAP and 2% FAP resulted in a remarkable increase in the broiler thymus index by 11.21% and 25.86%, respectively. Furthermore, the incorporation of 4% FAP exhibited a significant elevation of the bursa index by 26.06%, compared to the CON group. These results suggest that both FAP and UAP have the potential to improve the immune organ index of broilers, with FAP demonstrating more pronounced effects on these indexes. IgA and IgG, as primary immunoglobulins in serum, play vital roles in host defense against infections and are essential components of humoral immunity. Several studies explored the impact of *Astragalus* on immunoglobulin levels in animals. Hao et al. demonstrated that the inclusion of 15 g/kg *Astragalus* powder in the diet of fattening lambs significantly enhanced serum levels of IgA, IgG, and IgM [36]. Likewise, in a research by Xia et al. [37], dietary supplementation with *Astragalus* polysaccharide was found to stimulate the secretion of IgA and IgG in the serum of weaned rabbits. In the current study, only FAP2 group had significantly increased serum IgA and IgG levels in broilers at both 28 days and 42 days. In conclusion, compared to UAP, FAP exhibited a more significant positive impact on the immune function of broilers, indicating its potential as an effective immunomodulator for poultry.

Blood biochemical parameters serve as crucial indicators of the body’s nutrient function and metabolism [38]. Albumin (ALB), a crucial plasma protein, plays a pivotal role in maintaining the body’s nutritional status and regulating osmotic pressure [39]. Our results showed that ALB levels in the FAP2 group were significantly increased compared to the CON group at 28 d. ALT and AST are important indicators of liver function, with abnormal activity of these enzymes indicating liver injury [40]. However, in this study, there were no significant differences in ALT and AST levels among all groups. In addition, dietary supplementation with 4% FAP significantly increased the GLU levels of broilers at 28 days, which serves as a crucial monosaccharide for energy provision in animal blood. Within the normal range, there exists a positive correlation between sugar metabolism and GLU levels [41]. This suggests that dietary supplementation of 4% FAP enhances glucose metabolism in broilers. Meanwhile, this study revealed a significant impact on both ALB and GLU levels in young broilers, suggesting that FAP is more likely to affect serum parameters during the early stages of development. Creatinine (CREA) and urea (UREA) levels are commonly used to reflect the kidney function [42]. However, we found that supplemental feeding with FAP had no effect on serum CREA and UREA levels in broilers. Additionally, based on pathological observation of the liver and kidney, adding 1–4% FAP to the diet did not cause damage to these organs.

Oxidative stress is considered as an important factor influencing animal growth performance and predisposing animals to various diseases [43]. T-AOC represents the comprehensive capacity of a cell’s endogenous enzymatic and non-enzymatic systems to effectively eliminate free radicals [44]. GSH serves as the most important non-enzymatic antioxidant in the body, responsible for scavenging free radicals and preserving cell membrane integrity, making its content an important indicator of the body’s antioxidant capacity [45]. MDA is the main degradation product of lipid peroxidation, and its concentration can reflect the extent of oxidative damage [46]. The antioxidant properties attributed to *Astragalus* are mainly due to its active ingredients, such as polysaccharides, flavonoids, and saponins [47,48,49]. In our study, compared to the CON group, supplementation with 2% FAP significantly enhanced serum T-AOC levels and effectively reduced serum MDA levels in broilers at 28 d. Additionally, supplementation with 2% FAP resulted in a significant reduction in MDA levels in the serum at 42 d. However, supplementation with UAP did not show any effect on the antioxidant capacity of broilers at either 28 days or 42 days. These results suggest that 2% FAP exhibits better antioxidant activity than the same dose of UAP. A similar study was conducted by Lv et al. [50], who supplemented broiler diets with fermented Shenling Baizhu San and unfermented Shenling Baizhu San and noticed that the broilers fed with fermented Shenling Baizhu San showed greater antioxidant capacity. These results may be due to the fact that broilers absorb more of the active substance released during fermentation. Additionally, it is plausible that the fermentation process alters the structure of the active substance, thereby enhancing its antioxidant activity [15]. In summary, fermentation appears to promote the effectiveness of *Astragalus* application in broilers.

Intestinal microflora plays an important role in various physiological processes such as nutrient absorption, growth, immune regulation, and maintaining the biological barrier function of the host. Numerous studies have demonstrated the effectiveness of TCM in modulating the composition of intestinal microflora and promoting overall bodily health [51,52,53]. In this study, as the beneficial effects of FAP supplementation were mainly seen in the FAP2 group, we assessed the modulating effect of FAP and UAP on intestinal microflora in the CON, UAP2 and FAP2 groups. From the perspective of microflora structures, although the addition of UAP or FAP in the diet did not significantly affect the richness of cecum microflora, there was a tendency to increase the diversity of microflora, which was reflected by the increase in Shannon and Simpson indexes. Greater microbial diversity is associated with enhanced productivity, robust ecological communities, and heightened resistance to invasive species, thereby facilitating more efficient dietary utilization [54]. The results of β diversity in our study revealed significant differences in the microflora structures of the UAP and FAP groups compared to the CON group. It is possible that intensive farming weakens the influence of genetic factors on the intestinal microbial composition of broilers, making dietary and environmental factors more important [51]. In terms of intestinal microflora composition, *Firmicutes* and *Bacteroidetes* are the two most dominant phyla in the cecum of broilers. Changes in *Firmicutes* and *Bacteroidetes* can disrupt host homeostasis by altering energy acquisition, lipid metabolism, endocrine function, and inflammatory responses [55]. Healthy animals often maintain a dynamic balance in their intestinal microflora to ensure homeostasis. This study showed that 2% UAP and 2% FAP did not significantly affect the F/B. However, at the genus level, 2% FAP led to an increase in the relative abundance of *Alistipes*. Previous studies reported that *Alistipes* is capable of producing short-chain fatty acids, mitigating intestinal inflammation, and potentially exerting protective effects against various diseases such as liver fibrosis, colitis, cancer immunotherapy, and cardiovascular diseases [56]. Additionally, Wang et al. verified that glucan can substantially enhance the abundance of *Alistipes* and facilitate intestinal nourishment and defense [57]. Furthermore, the addition of 2% FAP and 2% UAP resulted in a reduction in the relative abundance of *Bacteroides*. Greiner and Bäckhed have proposed that compared to lean individuals, obese individuals exhibit decreased levels of *Bacteroides* in their gut microbiota [58]. Meanwhile, Xiang et al. found that *Bacteroides* is closely related to abdominal fat deposition in chickens and is more abundant in lean meat chickens, suggesting that the abundance of *Bacteroides* is negatively correlated with growth performance [59]. Therefore, our results indicate that feeding broilers with 2% FAP could potentially affect growth performance and immune function by modulating the composition of intestinal microflora.

## 5. Conclusions

The dietary supplementation of FAP has demonstrated significant improvements in the growth performance, immune function, antioxidant capacity, and regulation of intestinal flora in broilers. Based on these findings, FAP holds promise as a high-efficiency feed additive for broilers. It is recommended to incorporate an appropriate dosage of 1% to 2% FAP into broiler diets, with gradual increments corresponding to their age.

## Figures and Tables

**Table 1:** The main nutritive and active components of FAP and UAP.

Item	Nutritive Components (%)					Active Components (%)		
	CP	EE	CF	Ca	P	TP	TF	TS
FAP	9.62	0.8	21.8	0.23	0.27	5.05	0.25	0.58
UAP	8.87	0.7	26.4	0.26	0.3	3.66	0.16	0.48

FAP: fermented Astragalus powder; UAP: unfermented Astragalus powder; CP: crude protein; EE: ether extract; CF: crude fiber; Ca: calcium; P: phosphorus; TP: total polysaccharides; TF: total flavonoids; TS: total saponins.

**Table 2:** The ingredient and nutrient composition of the basal diet (% as fed basis).

Component	1–21 Days	22–42 Days
Ingredient (%)		
Corn	55.60	55.20
Soybean meal ^1^	29.00	24.00
Cottonseed meal	2.50	3.00
Wheat flour	4.00	4.00
Hydrolyzed feather meal	1.50	1.50
Dicalcium phosphate	0.90	0.80
Limestone powder	1.50	1.50
Bentonite	1.00	1.00
Soy oil	2.00	7.00
Premix ^2^	2.00	2.00
Total	100.00	100.00
Calculation of nutrients ^3^		
Metabolizable energy, kcal/kg	2894	3212
Crude protein, %	21.50	19.51
Calcium, %	0.96	0.84
Total phosphorus, %	0.66	0.55
Lysine, %	1.45	1.40
Methionine, %	0.54	0.50
Threonine, %	0.91	0.80

Note: ^1^ The crud protein content was 44%. ^2^ The premix provided the following per kilogram of diet: VA, 6000 IU;VD3, 2000 IU; VE, 30 mg; VK3, 2 mg; VB1, 3 mg; VB2, 5 mg; pantothenic acid, 800 mg; choline chloride 1500 mg; nicotinic acid, 30 mg; pyridoxine, 3 mg; folic acid, 500 mg; biotin, 0.2 mg; VBl2, 1 mg; Fe, 100 mg; Cu, 8 mg; Mn, 100 mg; Zn, 100 mg; I, 0.42 mg; Se, 0.3 mg. ^3^ The nutrient levels were calculated from date provided by Feed Database in China.

**Table 3:** Effect of dietary FAP supplementation on the growth performance in broilers.

Items	Treatments					SEM	*p*-Value
	CON	UAP2	FAP4	FAP2	FAP1		
Initial BW (g)	242.89	242.42	242.88	242.88	242.80	3.582	1.0
Final BW (g)	1291.58 ^c^	1324.74 ^bc^	1337.85 ^ab^	1374.15 ^a^	1373.83 ^a^	13.092	<0.001
14 to 42 days of age							
ADFI (g)	87.70	87.91	87.00	87.55	87.06	1.247	0.941
ADG (g)	37.45 ^c^	38.65 ^bc^	39.11 ^ab^	40.40 ^a^	40.39 ^a^	0.507	<0.001
F/G	2.34 ^a^	2.28 ^ab^	2.23 ^bc^	2.15 ^c^	2.16 ^c^	0.032	<0.001
14 to 28 days of age							
ADFI (g)	63.92	63.77	63.16	63.11	63.23	0.970	0.874
ADG (g)	33.14 ^c^	34.33 ^bc^	34.34 ^bc^	35.25 ^ab^	36.17 ^a^	0.446	0.001
F/G	1.93 ^a^	1.86 ^ab^	1.84 ^abc^	1.79 ^bc^	1.75 ^c^	0.032	<0.001
29 to 42 days of age							
ADFI (g)	111.27	112.05	110.83	111.93	110.89	2.148	0.936
ADG (g)	41.77 ^c^	42.98 ^bc^	43.87 ^abc^	45.56 ^a^	44.62 ^ab^	0.820	0.001
F/G	2.67 ^a^	2.61 ^ab^	2.53 ^abc^	2.46 ^c^	2.49 ^bc^	0.048	0.001
Survival rate (%)	93.75	96.88	96.88	98.44	98.44	3.125	0.565

^a–c^ Values with different superscripts in the same row differ significantly (*p* < 0.05) (*n* = 8). CON: control; UAP2, FAP4, FAP2, FAP1 represent that dietary supplementation of 2% unfermented *Astragalus* powder and 4%, 2%, 1% fermented *Astragalus* powder, respectively. ADG: average daily gain; ADFI: average daily feed intake; F/G: feed to gain ratio.

**Table 4:** Effect of dietary FAP supplementation on the main organ indexes in broilers.

Item	Treatments					SEM	*p*-Value
	CON	UAP2	FAP4	FAP2	FAP1		
Thymus index	3.21 ^c^	3.57 ^b^	3.87 ^ab^	4.04 ^a^	3.97 ^ab^	0.156	<0.001
Spleen index	1.51	1.54	1.54	1.60	1.58	0.710	0.724
Bursa index	1.42 ^b^	1.69 ^ab^	1.79 ^a^	1.74 ^ab^	1.74 ^ab^	0.120	0.029
Liver index	21.23	20.45	21.39	21.25	20.17	0.448	0.228
Kidney index	5.95	6.25	6.64	6.48	6.14	0.270	0.424

^a–c^ Values with different superscripts in the same row differ significantly (*p* < 0.05) (*n* = 8). CON: control; UAP2, FAP4, FAP2, FAP1 represent that dietary supplementation of 2% unfermented Astragalus powder and 4%, 2%, 1% fermented Astragalus powder, respectively.

**Table 5:** Effect of dietary FAP supplementation on the serum biochemical parameters in broilers.

Item	Treatments					SEM	*p*-Value
	CON	UAP2	FAP4	FAP2	FAP1		
28 days of age							
TP (g/L)	31.24	31.56	31.25	34.16	31.86	1.056	0.046
ALB (g/L)	11.54 ^b^	12.05 ^ab^	11.58 ^b^	12.71 ^a^	12.11 ^ab^	0.323	0.006
ALT (U/L)	4.06	4.29	4.84	4.18	4.44	0.618	0.754
AST (U/L)	186.75	182.00	184.50	185.63	193.63	10.436	0.843
GLU (mmol/L)	8.60 ^b^	9.13 ^ab^	10.43 ^a^	9.56 ^ab^	9.58 ^ab^	0.452	0.005
CREA (μmol/L)	45.68	46.36	47.38	45.91	47.16	2.36	0.936
UREA (mmol/L)	0.29	0.35	0.33	0.30	0.38	0.06	0.585
IgA (mg/mL)	0.25 ^b^	0.26 ^ab^	0.28 ^ab^	0.32 ^a^	0.30 ^ab^	0.010	0.020
IgG (mg/mL)	2.16	2.29	2.27	2.54	2.51	0.187	0.223
42 days of age							
TP (g/L)	33.31	33.25	34.00	34.69	34.09	1.118	0.685
ALB (g/L)	11.76	12.10	12.89	12.91	12.49	0.543	0.174
ALT (U/L)	4.79	4.86	4.64	4.88	4.56	0.704	0.989
AST (U/L)	190.00	195.38	199.13	203.75	201.25	9.782	0.662
GLU (mmol/L)	6.86	6.63	6.44	6.88	6.63	0.253	0.398
CREA (μmol/L)	44.66	43.00	45.66	46.19	45.14	2.37	0.714
UREA (mmol/L)	0.28	0.31	0.38	0.30	0.31	0.06	0.428
IgA (mg/mL)	0.30 ^b^	0.37 ^ab^	0.40 ^ab^	0.45 ^a^	0.36 ^ab^	0.474	0.036
IgG (mg/mL)	2.57 ^b^	2.89 ^ab^	3.17 ^ab^	3.44 ^a^	2.92 ^ab^	0.283	0.051

^a,b^ Values with different superscripts in the same row differ significantly (*p* < 0.05) (*n* = 8). CON: control; UAP2, FAP4, FAP2, FAP1 represent that dietary supplementation of 2% unfermented Astragalus powder and 4%, 2%, 1% fermented Astragalus powder, respectively. TP: total protein; ALB: albumin; ALT: alanine aminotransferase; AST: aspartate aminotransferase; CREA: areatinine; UREA: urea; GLU: glucose; IgA: Immunoglobulin A; IgG: Immunoglobulin G.

**Table 6:** Effect of dietary FAP supplementation on the antioxidant capacity in serum of broilers.

Items	Treatments					SEM	*p*-Value
	CON	UAP2	FAP4	FAP2	FAP1		
28 days of age							
T-AOC (mM)	0.52 ^b^	0.57 ^ab^	0.63 ^a^	0.64 ^a^	0.60 ^ab^	0.029	0.003
GSH (μmol/L)	37.94	39.23	40.77	41.45	40.69	2.087	0.464
MDA (nmol/mL)	5.77 ^a^	5.58 ^ab^	5.44 ^ab^	4.95 ^c^	5.10 ^bc^	0.167	<0.001
42 days of age							
T-AOC (mM)	0.46	0.49	0.51	0.53	0.48	0.038	0.398
GSH (μmol/L)	35.73	36.57	40.54	41.30	38.77	2.188	0.064
MDA (nmol/mL)	6.19 ^a^	5.95 ^ab^	5.80 ^ab^	5.55 ^b^	5.79 ^ab^	0.161	0.007

^a–c^ Values with different superscripts in the same row differ significantly (*p* < 0.05) (*n* = 8). CON: control; UAP2, FAP4, FAP2, FAP1 represent that dietary supplementation of 2% unfermented Astragalus powder and 4%, 2%, 1% fermented Astragalus powder, respectively. T-AOC: total antioxidant capacity; GSH: glutathione; MDA: malondialdehyde.

## Data Availability

Research data are available upon request to the authors.
